# Effect of neutrophil depletion on gelatinase expression, edema formation and hemorrhagic transformation after focal ischemic stroke

**DOI:** 10.1186/1471-2202-6-49

**Published:** 2005-08-03

**Authors:** Alex K Harris, Adviye Ergul, Anna Kozak, Livia S Machado, Maribeth H Johnson, Susan C Fagan

**Affiliations:** 1 Program in Clinical and Experimental Therapeutics, College of Pharmacy, University of Georgia, Augusta, Georgia, USA; 2 Vascular Biology Center, Medical College of Georgia, Augusta, Georgia, USA; 3 Veteran's Affairs Medical Center, Medical College of Georgia, Augusta, Georgia, USA; 4 Department of Biostatistics, Medical College of Georgia, Augusta, Georgia, USA; 5 Department of Neurology, Medical College of Georgia, Augusta, Georgia, USA

## Abstract

**Background:**

While gelatinase (MMP-2 and -9) activity is increased after focal ischemia/reperfusion injury in the brain, the relative contribution of neutrophils to the MMP activity and to the development of hemorrhagic transformation remains unknown.

**Results:**

Anti-PMN treatment caused successful depletion of neutrophils in treated animals. There was no difference in either infarct volume or hemorrhage between control and PMN depleted animals. While there were significant increases in gelatinase (MMP-2 and MMP-9) expression and activity and edema formation associated with ischemia, neutrophil depletion failed to cause any change.

**Conclusion:**

The main finding of this study is that, in the absence of circulating neutrophils, MMP-2 and MMP-9 expression and activity are still up-regulated following focal cerebral ischemia. Additionally, neutrophil depletion had no influence on indicators of ischemic brain damage including edema, hemorrhage, and infarct size. These findings indicate that, at least acutely, neutrophils are not a significant contributor of gelatinase activity associated with acute neurovascular damage after stroke.

## Background

The matrix metalloproteinases (MMPs) are a family of some 23 zinc dependent proteases that, collectively, possess the ability to degrade nearly every component of the extra-cellular matrix [1-3]. The activity of the MMPs is tightly controlled through proteolytic activation of the zymogen forms and stoichiometric binding of tissue inhibitors of metalloproteinases (TIMPs). The MMPs play an important role in many physiological processes due to their inherent ability to remodel tissues [2,3]. However, in disease states such as vascular disease and stroke, the MMPs may become deleterious due to dysregulation and can result in tissue injury and inflammation. Specifically, the MMPs may be involved in the degradation of the basal lamina in reperfusion injury resulting in disruption of the blood brain barrier and hemorrhagic transformation [4].

Recently, several lines of evidence have demonstrated the involvement of the MMPs in cerebral ischemia. Studies in rat, mouse, and baboon models have shown that MMP-9 is up-regulated following transient focal ischemia [5-8]. Additionally, Asahi et al. have shown that MMP-9 knockout as well as MMP-9 inhibition reduces ischemic lesion volume [9]. However, others have shown that pharmacological inhibition of MMP-9 yields no change [10,11]. Lapchak et al. demonstrated that broad-spectrum MMP inhibition (BB-94) reduced the incidence of hemorrhage in tPA treated brains when administered shortly after the onset of ischemia [11]. In addition, Sumii et al. were able to show a reduction in hemorrhage severity in tPA treated animals given the same MMP inhibitor BB-94 [10].

Clearly the MMPs are involved in the pathology of cerebral ischemia and hemorrhagic transformation. However, there is still uncertainty as to the origin of MMP activity. Immunohistochemical studies from Asahi et al. demonstrated that MMP-9 is primarily up-regulated in the vascular spaces while others have shown that there is a concomitant staining of neutrophils in the areas of MMP-9 activation suggesting a role for the neutrophil in the up-regulation of MMP-9 [12-14]. Indeed, a recent study has shown that prevention of neutrophil infiltration significantly reduces MMP-9 up-regulation in an occlusion/reperfusion model of ischemia [15]. The source of MMP activity in focal cerebral ischemia is important to the development of therapies to target this mediator of neurovascular injury. It is still unknown whether neutrophils are an important source of MMPs in experimental hemorrhagic transformation.

The objective of the current study was to evaluate the role of the neutrophil in hemorrhagic transformation and edema formation in a hyperacute 3-hour occlusion/reperfusion model of focal ischemia. The central hypothesis was that depletion of neutrophils would reduce hemorrhage development due to prevention of the up-regulation of MMP-9.

## Results

### Neutrophil depletion

**Figure 1:**
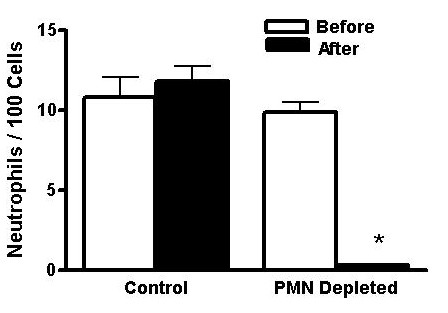
Neutrophil counts at baseline and at 24 hours after normal rabbit serum (control) or anti-PMN antibody (PMN-depleted) administration. Anti-PMN antibody resulted in a consistent decrease in circulating neutrophils to less than 10% of baseline counts whereas there was no change in neutrophil counts in control animals that received the normal rabbit serum. *p < 0.001 control (n = 8) vs after PMN-depletion (n = 10).

Control and neutrophil-depletion groups received normal rabbit serum and anti-PMN antibody, respectively, 24 hours prior to middle cerebral artery occlusion (MCAO) surgery. The dose of the anti-neutrophil antibody chosen (and this was batch-specific in our preliminary studies – not shown) was very effective in reducing the circulating neutrophils. In control animals that received normal serum, neutrophil count, expressed as mean ± SE of percent of total leukocyte count, was 11.8 ± 0.92% and as expected did not differ from baseline. In the depletion group, administration of anti-PMN antibody reduced the neutrophil count to 0.3 ± 0.11% from 9.4% at baseline (p < 0.0001 compared to serum). All animals included in the neutropenic group had >90% depletion of their neutrophils prior to the stroke surgery, as assessed by a blinded investigator using a hemocytometer (Fig. 1). The body weight of control animals was 288 ± 4 g at baseline, 292 ± 4 g prior to stroke and 237 ± 4 g at 24 hours. In the antibody-treated group, body weight was 300 ± 4 g at baseline, 292 ± 5 prior to stroke surgery, and 240 ± 5 g at 24 hours.

### Gelatinase quantification

**Figure 2:**
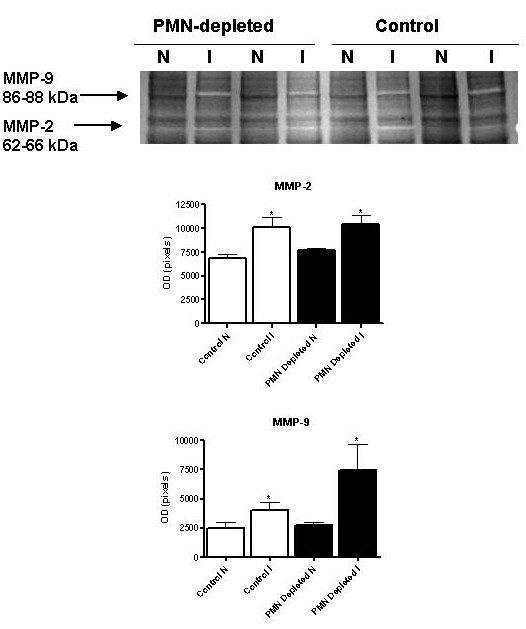
MMP activity is increased in focal cerebral ischemia. (A) Representative zymogram showing changes in MMP activity in the ischemic (I) and non-ischemic (N) hemispheres. (B) Densitometric analysis of lytic bands indicates increases in MMP-2 and MMP-9 activity in ischemic hemispheres from control and PMN-depleted animals. Anti-PMN antibody treatment has no affect on MMP activity. (*p < 0.05 vs contralateral hemisphere, control n = 6, PMN n = 4).

**Figure 3:**
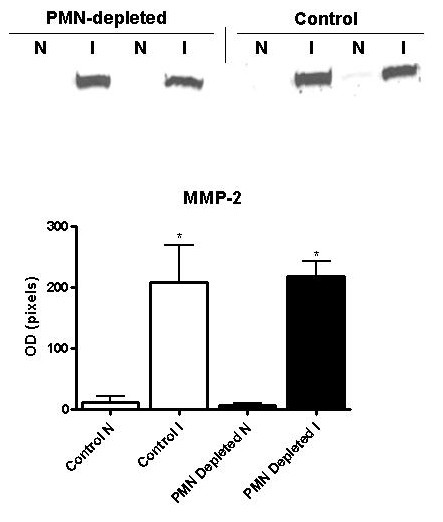
MMP-2 protein expression is increased in ischemia. (A). Representative immunoblot demonstrating changes in MMP-2 protein expression in ischemic hemispheres from control and PMN-depleted animals. (B) Densitometric analysis of immunoreactive bands indicates that MMP-2 protein is increased in ischemia and is unchanged by neutrophil depletion. (*p < 0.05 vs contralateral hemisphere, saline n = 6, PMN n = 4).

**Figure 4:**
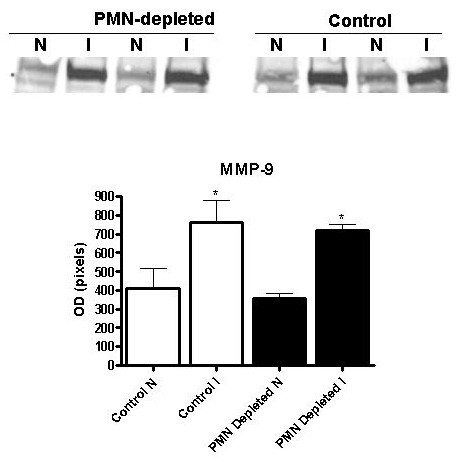
MMP-9 protein expression is unchanged by neutrophil depletion. (A). Representative immunoblot showing immunoreactive MMP-9 bands in both ischemic (I) and non-ischemic (N) hemispheres. (B) Densitometric analysis indicates an ischemia induced elevation of MMP-9 expression that is unaffected by PMN depletion. (*p < 0.05 vs contralateral hemisphere, control n = 6, PMN n = 4).

Ischemia for three hours followed by 24 h reperfusion resulted in a significant increase in the expression and activity of both MMP-2 and MMP-9 in the ipsilateral hemispheres (Fig. 2). Quantitative analysis of the gelatin zymography revealed consistent increases in the lower molecular weight form of MMP-9 (88 kDa). Although there was a faint band corresponding to 95 kDa MMP, the gelatinolytic activity in control vs neutrophil-depleted animals was similar. Protein levels of MMP-2 (Fig. 3) and MMP-9 (Fig. 4) were significantly increased in the ischemic hemisphere and neutrophil depletion did not affect MMP protein levels.

### Infarct size, edema and hemorrhage assessment

**Figure 5:**
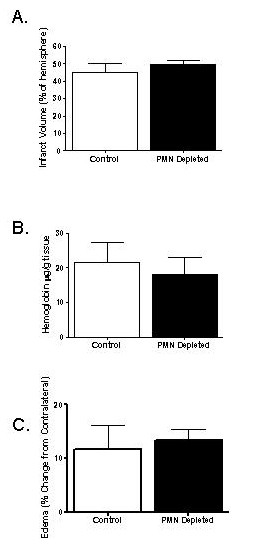
Infarct size, edema formation and hemoglobin excess differences between control and PMN-depleted animals. (A). Infarct size, as measured by TTC staining, indicated no differences between depletion and control groups. (B). Likewise, PMN depletion had no affect on the development of hemorrhagic transformation. (C) Edema determined as the difference in area between the ischemic and non-ischemic hemispheres was similar in neutrophil-depleted and control animals (n = 8 control, n = 10 PMN).

There was no significant effect of neutrophil depletion on either infarct size or the development of hemorrhagic transformation in our model (Figure 5). In addition, cerebral edema formation was similar between untreated and neutrophil-depleted animals. Neurologic examination at 24 hours was not significantly different between the two treatment groups (not shown).

## Discussion

Cerebral ischemia and reperfusion are known to induce large increases in MMP-9 protein and activity in the affected hemisphere and MMP-9 has been associated with hemorrhage formation in humans [16] and experimental animals [10]. Experimental inhibition of the MMPs has been associated with decreased hemorrhage formation and improved stroke outcome [11]. Since neutrophils are a known source of MMP-9, this study was designed to determine whether neutrophil depletion, prior to cerebral artery occlusion, would decrease MMP expression and reduce hemorrhagic transformation.

Despite the fact that neutrophil depletion was essentially complete prior to the focal cerebral ischemia in our experiment, we detected an increase in gelatinolytic activity corresponding to 86–88 kDa MMP-9. There was a faint band for the 95 kDa proMMP-9, and the activity did not differ between the ischemic and non-ischemic hemispheres. Immunoblotting experiments confirmed the results of gelatin zymography and showed that specific up-regulation of the MMP-9 protein is responsible for increased gelatinolytic activity. Justicia et al. reported that, in a 1 h MCAO model in which the neutrophil depletion or antagonism was achieved by vinblastine administration or neutralizing antibodies against VCAM-1, respectively, there was no effect on the 88 kDa MMP-9 expression but, increases in 95 kDa MMP-9 activity were prevented. Our results are in agreement with this observation that neutrophils are not the source of 88 kDa MMP-9. However, we do not detect any increase in 95 kDa MMP-9. This difference may be due to the duration of ischemia (1 h vs 3 h) and the extent of ischemic damage [15]. We have previously shown that hemorrhagic transformation occurs in various different regions of the injured hemisphere including: preoptic area, striatum and the lateral cortex in this model and that development of hemorrhagic transformation is related to the duration of occlusion, with no hemorrhage when reperfusion occurs after only 1 hour [17,18].

In this model of focal cerebral ischemia and hemorrhagic transformation, where the MCA was occluded for 3 hours prior to reperfusion, no significant effect of neutrophil depletion could be shown on either neurovascular damage or neurologic function. Also, from our results, it is clear that neutrophils are not an important contribution to the increased MMP-9 expression in the 24 hours after this injury. It is possible that the contributions of neutrophils are greater when the injury to the brain is less profound than in our model. Several different investigators have demonstrated that preventing the adhesion of neutrophils [19] after ischemia and reperfusion reduces the ultimate injury. Maier et al. reported that in superoxide dismutase (SOD) knock-out animals that are more susceptible to ischemic damage, neutrophils are not the source of MMP-9 contributing to blood brain barrier breakdown providing further support for the results of this present study [20].

Activated neutrophils release free radicals and proteolytic enzymes such as MMP-8 (neutrophil elastase), in addition to activating cytokines, which further the recruitment of leukocytes to the site of injury. We initially thought that neutrophil adherence and activation may be an early contributor to the development of microvascular injury and hemorrhagic transformation after cerebral ischemia but this turned out to be not the case [21]. It is still possible that neutrophils are involved in the destruction of the basal lamina, but it is likely to occur later in the process, after neutrophil recruitment is maximal.

## Conclusion

Although neutrophils have been shown to contain MMP-9, release of MMP-9 by neutrophils is probably not the mechanism of the early microvascular damage leading to edema formation and hemorrhagic transformation in our model. In addition, neutrophil adhesion is not necessary for the increase in activation of MMP-9 after ischemia. Although neutrophils may contribute to the ultimate degree of neurologic damage after ischemia and reperfusion in the brain, they are not necessary for the development of hemorrhagic transformation.

## Methods

### Neutrophil depletion

Neutrophil depletion was accomplished by intravenous administration of a polyclonal rabbit anti-rat polymorphonuclear neutrophil (PMN) antiserum (Accurate Chemical and Scientific Corporation, Westbury, NY) 24 hours prior to ischemia [22]. Animals were randomized to receive either the PMN antiserum (0.3 mL diluted in 1 mL saline) or an equal volume of normal rabbit serum (control) 24 hours prior to surgery. Neutrophil counts, as a percentage of total leukocyte count, were determined at baseline and 23 hours post administration to determine the level of depletion.

### Animal preparation/physiological monitoring

The Care of Experimental Animal Committee of Medical College of Georgia approved the protocol. Male Wistar rats, from the Charles River Breeding Company (Wilmington, MA, USA) within a narrow range of body weight (250–300 g) were used. All animals were anesthetized with 2% isoflurane via inhalation. Cerebral ischemia was induced using the intraluminal suture MCAO model [23]. The MCA was occluded with a 19–21 mm 4-0 surgical nylon filament, which was introduced from the external carotid artery lumen into the internal carotid artery to block the origin of the MCA. The suture was removed after 3 hours of occlusion. We have shown previously that hemorrhagic transformation is related to the duration of occlusion in this model [17,18]. Prior to, and after reperfusion, each rat was evaluated neurologically using the Bederson Scale [24]. After 21 hours of reperfusion, the animals were anesthetized with ketamine 44 mg/kg and xylazine 13 mg/kg I.M. (cocktail), perfused with saline, sacrificed, and the brain tissue was removed.

### Brain preparation and homogenization

The brains were sliced into seven 2-mm thick slices in the coronal plane and stained with a 2% solution of 2, 3, 5-triphenyltetrazolium chloride (TTC) (Sigma Chemical Co., USA) for 15–20 minutes. Images of the stained sections were taken. Grossly visible infarction zones were quantified using image analysis software (Zeiss- KS 300) in *pixel*^*2 *^[25]. Because brain edema can affect the accuracy of infarct assessment [26], the measurement of the corrected infarct size was taken [27,28]. The ischemic and non-ischemic hemispheres of the slices for the ELISA assay were separated and processed individually, using the non-ischemic side as a control. Each slice was homogenized, vortexed and sonicated. Quantification of the hemoglobin was accomplished using a direct ELISA method, as previously described [29].

For zymography and western blotting, brains were placed in a chilled coronal matrix and 2-mm sections were removed from the core of the infarct. The slices were collectively placed, by hemisphere, into labeled tubes, flash frozen, and stored at -80°C until use. Samples were homogenized according to the methods of Heo et al. [7]. Briefly, samples were placed in 300 μL cold working buffer consisting of 50 mmol/L Tris-HCl (pH 7.5), 75 mmol/L NaCl, and 1 mmol/L PMSF, homogenized with a glass homogenizer and centrifuged at 4°C for 20 minutes at 9000 rpm. The supernatant was collected and stored at -80°C until use. Protein concentrations were determined using the Bradford method.

### Gelatin zymography

On the day of the experiment, samples (20 μg protein/sample) were loaded onto 10% gelatin zymogram gels (Bio-Rad) and separated under nonreducing conditions [30]. The gels were then rinsed twice in 2.5% Triton X-100 and incubated for 24 hours in substrate buffer containing 21 mM Tris·HCl, 10 mM CaCl_2_, 0.04% NaN_3_. Gels were then stained by Coomassie blue R-250 followed by destaining in 55% methanol and 7% acetic acid. Lytic activity was viewed as clear bands on a dark blue background and was quantitated by densitometric analysis (Gel-Pro v 3.1, Media Cybernetics, Carlsbad, CA.).

### Edema quantitation

Brain edema was measured as a difference in area between the ischemic and non-ischemic hemispheres. The brain section analyzed was the one corresponding to the area of the ischemic damage in four consecutive 2-mm slides. The brain slides were analyzed separately and the areas and differences combined for whole hemi-section comparison. The area analysis was done using Zeiss KS-300.

### Statistics

A Wilcoxon rank sum test was performed to make group comparisons (control vs. depletion) in MMP activity and protein expression for the difference between the ischemic and non-ischemic sides of the brain and group comparisons for neutrophil depletion, edema, infarct size and hemorrhagic transformation. A Wilcoxon signed rank test was used to make within rat comparisons between ischemic and non-ischemic sides of the brain for MMP activity and protein expression. Statistical significance was determined at p < 0.05 and SAS 8.2 was used for all analyses.

## Authors' contributions

AKH performed all the zymography and western blotting and contributed to preparation of the manuscript.

AE contributed to the optimization of zymograms, data interpretation and preparation of the manuscript.

AK did all the animal preparation, MCAO, infarct and hemoglobin analysis.

LM completed the edema analysis and contributed to the manuscript preparation.

MHJ performed the statistical analysis.

SCF coordinated all aspects of this work and prepared the manuscript.

All authors approved the final version of the manuscript.
